# Quality of Evidence Supporting the Effects of Tai Chi Exercise on Essential Hypertension: An Overview of Systematic Reviews and Meta-Analyses

**DOI:** 10.1155/2022/4891729

**Published:** 2022-04-30

**Authors:** Hongshuo Shi, Zixuan Wu, Dan Wang, Chengda Dong, Pulin Liu, Guomin Si, Ting Liu

**Affiliations:** ^1^Shandong University of Traditional Chinese Medicine, Jinan, China; ^2^Guangzhou University of Chinese Medicine, Guangzhou, China; ^3^Shandong Provincial Hospital Affiliated to Shandong First Medical University, Jinan, China

## Abstract

**Objectives:**

Tai Chi (TC) is a potential complementary treatment for essential hypertension (EH). This overview systematically summarizes and evaluates the existing evidence of TC in the therapy of EH.

**Methods:**

Systematic reviews (SRs)/meta-analyses (MAs) on TC interventions for EH were comprehensively searched in seven databases. Methodological quality, risk of bias, reporting quality, and quality of evidence were assessed by means of the Assessment of Multiple Systematic Reviews 2 (AMSTAR-2), the Risk of Bias in Systematic (ROBIS) scale, the list of Preferred Reporting Items for Systematic Reviews and Meta-Analysis (PRISMA), as well as the Grading of Recommendations Assessment, Development, and Evaluation (GRADE) system.

**Results:**

Twelve published SRs/MAs were included in our study. According to the results of the AMSTAR-2, ROBIS, PRISMA, and GRADE assessment, only 1 SR/MA was assessed as high quality and only 1 SR/MA was assessed as low risk of bias. Only 2 SRs/MAs have been fully reported on the checklist. In addition to that, the quality of evidence was assessed for a total of 69 outcome indicators extracted from the SRs/MAs included in this overview, and only 3 items were assessed as high quality.

**Conclusions:**

TC may be an effective and safe complementary treatment for EH. However, this conclusion must be approached with caution, as the quality of the evidence provided by the SRs/MAs is usually low.

## 1. Introduction

Cardiovascular disease accounts for one-third of global deaths and remains a growing public health concern [1]. As one of the most common diseases in the world, hypertension (HT) is the most important risk factor for cardiovascular disease [2]. HT affects more than 1.39 billion people worldwide and is estimated to cause 9.4 million deaths each year, making HT one of the most serious chronic problems threatening public health [3]. HT is divided into essential hypertension (EH) and secondary HT, of which the former is the most common (about 90%), but its pathogenesis is still unclear [4]. Although the treatment of HT has been continuously explored in the past few decades, HT drug therapy is still used as the main means of treatment of EH [5]. The likelihood that patients with EH will be able to maintain their blood pressure in the normal range with antihypertensive medications alone remains low [6]. As a result, many patients have to take several antihypertensive drugs at the same time to control their blood pressure. However, this practice can impose an increased financial burden and may have unforeseen side effects [7]. Therefore, there is an urgent need for better management of HT.

The Canadian Hypertension Education Program (CHEP), the Eighth Joint National Committee (JNC-8), and the American Heart Association (AHA) have recommended aerobic exercises for people with HT [8], and Traditional Chinese Exercise (TCE) has gained worldwide popularity due to its beneficial effects on improving the physical and mental health of patients with chronic diseases [9]. Tai Chi (TC) is a traditional Chinese physical and mental exercise with moderate movement intensity. Originated in China thousands of years ago, TC combines Chinese philosophy with martial arts and healing arts [10]. A growing number of studies have shown that TC can modulate pressure receptors in the carotid sinus and aortic arch, and play a part in dilating coronary arteries and lowering blood pressure [11, 12].

Many systematic reviews/meta-analyses (SRs/MAs) have been conducted to evaluate the potential therapeutic benefits of TC for patients with EH. However, the conclusions are inconsistent due to the defects of the quality and the method of the preliminary research. The overview of systematic reviews is a novel tool for solving specific and key issues related to policies and practices [13]. The purpose is to synthesize the evidence from multiple SRs/MAs into a useable document that can be employed to guide healthcare professionals and decision-makers. To this end, our research is to use a systematic overview to critically evaluate the scientific quality of related SRs/MAs in the TC treatment of EH.

## 2. Methods

### 2.1. Research Methods

The SRs/MAs overview was based on the guidelines specified in Cochrane Handbook [14] and relevant methodologies for a high-quality overview [15].

### 2.2. Development of Inclusion and Exclusion Criteria

#### 2.2.1. Literature Inclusion Criteria

- Type of research: This overview included SRs/MAs of randomized controlled trials (RCTs) on the effects of TC exercise on EH.
- Type of participants: The subjects were patients diagnosed with EH by any international [16] or national [17] standard regardless of their gender, age, or race.
- Type of intervention: The intervention for the control group was antihypertensive drugs (AHD), other exercises (OE), no treatment (NT), routine care (RC), or health education (HE), and the intervention for the experimental group was TC exercise or TC combined with the treatments received by the control group.
- Types of outcomes: Outcomes assessed in this overview included systolic/diastolic blood pressure (S/DBP), total cholesterol (TCL), high-density lipoprotein (HDL), triglycerides (TG), low-density lipoprotein (LDL), quality of life (QOL), and body mass index (BMI).

#### 2.2.2. Exclusion Criteria

Repeated publications, other overviews, network meta-analyses, narrative reviews, dissertations, and conference abstracts were excluded.

### 2.3. Data Sources and Search Strategy

Seven electronic databases were searched by 2 researchers (HS-S and D-W) from their respective inception times to January 1, 2022, including the Cochrane Library, PubMed, EMBASE, China Biomedicine (CBM), Wanfang Database, CNKI, and Chongqing VIP. The literature search was carried out using a combination of MeSH terms and free words, and MeSH terms include “Tai Chi,” “Hypertension,” “Systematic Review,” and “Meta-Analysis,” and adjustment was made according to different databases. The search strategy of the PubMed database is shown in Table 1.

### 2.4. Literature Screening and Data Extraction

Two researchers (HS-S and ZX-W) independently screened the retrieved literature studies. Then, the researchers removed the duplicate publications, read the publication titles and abstracts, and finally read the full text to assess their eligibility. All SRs/MAs were read by two independent researchers (D-W and CD-D), and the following data were extracted from the SRs/MAs: first author, publication year, country, number of RCTs included, interventions for experimental and control groups, included RCT quality assessment tools, and main conclusion. The disagreement between the two researchers was resolved through discussion.

### 2.5. SRs/MAs Quality Estimate

Two researchers (PL-L and HS-S) independently assessed the methodological and evidence quality of the included MAs.

#### 2.5.1. Estimate of Methodological Quality

The methodological quality of the included SRs/MAs was assessed by the Assessment System for Evaluating Methodological Quality 2 (AMSTAR-2) [18]. Seven (2, 4, 7, 9, 11, 13, and 15) of the 16 items in the tool were critical areas.

#### 2.5.2. Estimate of Risk of Bias

The Risk of Bias In Systematic Review (ROBIS) [19] scale was used in this overview to evaluate the risk of bias for the inclusion of SRs/MAs. The scale was divided into three stages to assess the overall risk of bias of the included SRs/MAs.

#### 2.5.3. Estimate of Reporting Quality

The quality of each SR/MA report of the included SRs/MAs was evaluated by the list of Preferred Reporting Items for Systematic Reviews and Meta-Analyses (PRISMA) [20], which consisted of 27 items focusing on reporting methods and results that were incorporated into SRs/MAs.

#### 2.5.4. Assessment of Quality of Evidence

The quality of evidence for each SR/MA outcome was evaluated by means of the Grading of Recommendations Assessment, Development, and Evaluation (GRADE) [21], according to which, five aspects will lead to the degradation of evidence quality, including limitations, inconsistencies, indirectness, imprecision, and publication bias.

## 3. Results

### 3.1. Results on Literature Search and Selection

**Figure 1:**
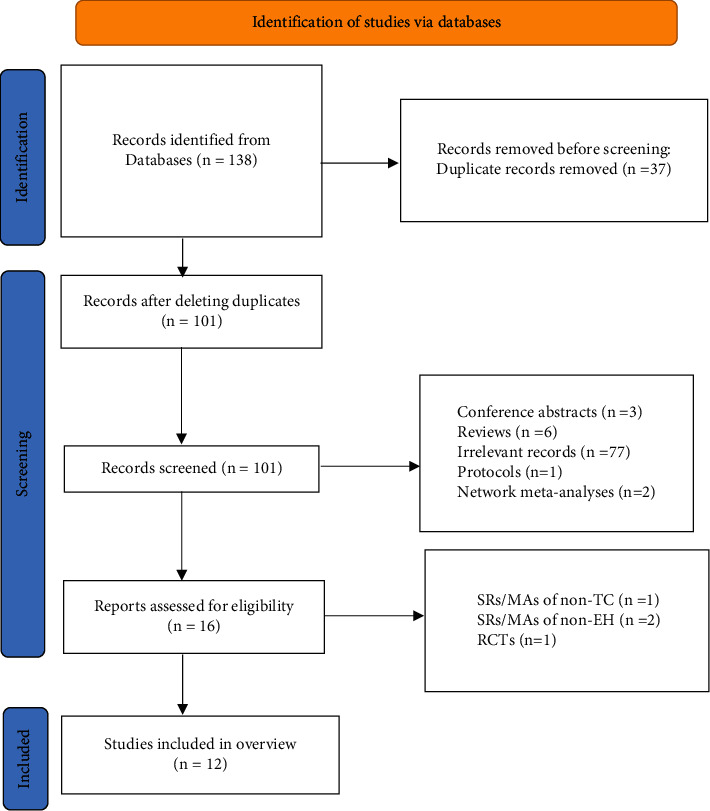
The flowchart of the screening process.

Through our search strategy, a total of 138 articles were identified. After removing 37 duplicate articles, the researchers screened the remaining 101 articles by reading the titles and abstracts. Subsequently, 16 articles were obtained. After reading the full text, 1 article [22] was not about SRs/MAs in TC, and 2 SRs/MAs [23, 24] were not about people with EH. In addition, there was an article [25] on an RCT of the efficacy of TC in EH. Finally, a total of 12 SRs/MAs [26–37] were finally included in this overview. The process of study selection is shown in Figure 1.

### 3.2. Description of Included SRs/MAs

The characteristics included in the overview are shown in Table 2. These SRs/MAs were all published between 2011 and 2021, 7 [26–32] of which were in English, and the remaining 5 [33–37] were in Chinese, and all were written by Chinese authors. The SRs/MAs included in this overview contained a total of 58 RCTs, of which 42 (72.4%) RCTs are overlapping (Table 3). The number of RCTs was between 5 and 28, and the sample size was between 402 and 2,937. In terms of quality evaluation scales, 9 SRs/MAs [26–33, 37] used the Cochrane risk of bias standard, and 3 SRs/MAs [34–36] used the Jadad Scale.

### 3.3. Results on SRs/MAs Quality Assessment

#### 3.3.1. Methodological Quality Assessment

The evaluation details of the included MAs on the AMSTAR-2 are shown in Table 4. Only 1 SR/MA [26] was rated as high quality, and the quality of the remaining SRs/MAs [27–37] was rated very low since more than one critical area was missing. Methodological quality limitations come from the following items: Item 2 (only 3 SRs/MAs registered the study protocol), Item 7 (only 1 SR/MA [26] provided a literature exclusion list), and Item 13 (when interpreting the evaluation results, only 4 SRs/MAs [26, 28, 29, 36] considered the risk of bias in the main study).

#### 3.3.2. Risk of Bias of the Included SRs/MAs

Regarding the results of the ROBIS assessment, Phase 1 assessed the relevance of the study topic and Domain 1, with all MAs rated as low risk of bias in both items. In Domain 2, 6 SRs/MAs [26–28, 30, 32, 33] were assessed as low risk. In Domain 3, 7 SRs/MAs [26, 27, 29, 30, 32, 34, 37] were assessed as low risk of bias and 3 SRs/MAs [26, 29, 34] were assessed as low risk of bias in Domain 4. In Phase 3, only 1 SR/MA [26] had a low risk of bias. The evaluation details of the included SRs/MAs on the ROBIS scale are shown in Table 5.

#### 3.3.3. Report Quality of the Included SRs/MAs

The results of the PRISMA assessment are shown in Table 6. Twenty-one of the 27 items had a “yes” response rate of over 70%, indicating the inclusion of relatively complete reporting of SRs/MAs. Nevertheless, there are reporting deficiencies on some items. The reports of Item 5 (protocol and registration) and Item 8 (search) were incomplete (the “yes” response rate was less than 50%).

#### 3.3.4. Evidence Quality of the Included SRs/MAs

The 12 SRs/MAs included 69 outcome indicators related to the effectiveness of TC for EH. By means of GRADE evaluation, 3 were rated as high quality of evidence, 15 moderate, 29 low, and 22 very low for all the outcome indicators. Inconsistency (*n* = 45) and publication bias (*n* = 45) were the most common downgrading factors, followed by the risk of bias (*n* = 36), imprecision (*n* = 21), and indirectness (*n* = 0). GRADE specific assessment details are shown in Table 7.

### 3.4. Summary Results of the Included SRs/MAs

The result indicators extracted from the included studies are listed in Table 7.

#### 3.4.1. Blood Pressure

Fifteen SBP-related outcomes were reported in 11 SRs/MAs [26–29, 31–37], all of which indicated that TC was effective in reducing SBP in EH patients. Of the 11 SRs/MAs [26–29, 31–37] that reported 16 outcomes related to DBP, only 1 [33] outcome showed that TC was ineffective in reducing DBP compared with HE/NT and the rest showed that TC was effective in reducing DBP.

#### 3.4.2. Outcomes Related to Lipid Metabolism

Three SRs/MAs [26, 27, 31] reported the effect of TC on TCL, and the results indicated that TC could effectively reduce TCL in EH patients. Four outcome indicators in three SRs/MAs [26, 27, 31] reported the effect of TC on TG, 3 outcome indicators [26, 27, 31] showed that TC could effectively reduce TG, and 1 outcome indicator [26] showed that TC was ineffective in reducing TG in EH patients compared with HE/NT. Three SRs/MAs [26, 27, 31] reported that TC was ineffective in improving HDL in EH patients. In addition, three SRs/MAs [26, 27, 31] reported that TC could reduce LDL in EH patients.

#### 3.4.3. Other Outcome Measures

Three SRs/MAs [27, 29, 30] reported that TC exercise could improve QOL in EH patients. Three SRs/MAs [29, 31, 32] reported the efficacy of TC exercise on BMI, and 2 SRs/MAs [29, 32] showed that TC could reduce BMI in EH patients. Two SRs/MAs [28, 35] reported the superiority of TC exercise in terms of efficacy in treating EH patients. The results of two SRs/MAs [31, 32] indicated that TC could reduce WC in EH patients. In addition to this, one SR/MA [27] showed that TC exercise reduced blood glucose levels in EH patients.

#### 3.4.4. Adverse Event

The five SRs/MAs [26, 28, 29, 31, 32] described TC as having a good safety profile.

## 4. Discussion

HT is an important risk factor for a variety of cardiovascular and cerebrovascular diseases. With the development of science and technology, people gradually realize the important role of TC in healthcare as well as the prevention and treatment of cardiovascular and cerebrovascular diseases [38]. In recent years, multiple SRs/MAs have been performed to elucidate the potential efficacy and safety of TC on EH. Therefore, we conducted this overview to synthesize multiple published SRs/MAs to assess their methodological quality and level of evidence.

### 4.1. Summary of the Main Findings

This overview included 12 SRs/MAs on the impact of TC on EH. All SRs/MAs were based on RCTs and published from 2011 to 2021. Among them, 9 (9/12, 75%) SRs/MAs were published in the past five years, indicating that the improvement effect of TC on EH has attracted increasing attention over the period. We performed an extraction analysis for all the original RCTs covered by the SRs/MAs included in this overview, and we found differences in the inclusion of RCTs across these SRs/MAs. The reasons are as follows: (1) the search date and the number of RCTs included in the earliest published SRs/MAs were limited; (2) the focus on the outcomes was different in the included SRs/MAs, e.g., Song 2021 [30] focused on the quality of life of EH patients; (3) the age limit of the included population varied greatly, e.g., Jin 2018 [34] focused on middle-aged and elderly patients with EH; (4) The trial period of RCTs was different, e.g., Liang 2020 [27] limited the trial period of RCTs to more than one month.

Based on the results of the AMSTAR-2 evaluation in this overview, the methodological quality of only 1 included SR/MA was rated high and that of the remaining SRs/MAs was rated very low, especially in Item 2 (Protocol registration, 3/12, 25%), Item 7 (Exclusion list, 1/12, 8.3%), and Item 13 (RoB account, 4/12, 33.3%). Only 3 SRs/MAs contained initial research protocol registrations, which could lead to greater than expected adjustments to the research process, increasing the risk of bias and impacting the rigor and credibility of the final MAs results [39]. Only 1 SR/MA provided a complete exclusion of the reasons for each study, which may affect the reliability of the results and assessment of publication bias. The provision of a list of exclusion researches can more strongly demonstrate the rigor of the literature screening process. The authors of the 8 SRs/MAs did not consider the risk of bias of the included RCTs when interpreting or discussing the study results, which may reduce the reliability of the final results. The ROBIS scale was used to assess the risk of bias of the included SRs/MAs. Among the included SRs/MAs, only one SR/MA was rated as low risk, and the remaining lacked a reasonable explanation for the risk of bias, which affected the quality of SRs/MAs and reduced the utility of evidence. Similar to the results of the AMSTAR-2 assessments, the PRISMA assessment results indicated a lack of registration of the programs. In addition, only search keywords were provided but no specific search strategies were provided, which reduced the reproducibility and credibility of the research.

Based on the GRADE assessment for the 69 outcome indicators, 3 were rated as high, 15 moderate, 29 low, and 22 very low in terms of the evidence quality. The main downgrading factors were inconsistency, publication bias, and risk of bias. Further analysis revealed high inconsistency in many outcomes, possibly due to the large clinical and methodological differences in the included RCTs, such as the duration, frequency, and pattern of TC exercise that varied widely across these studies. Besides, the absence of an assessment of publication bias also led to the downgrading of the quality of the evidence, which affected the confidence of the results. In addition to this, the risk of bias was also an important factor that lowered the quality of evidence, implying that the quality of the RCTs included in the SRs/MAs was low. According to the assessment of the methodological quality of included RCTs, most only referred to randomization without providing a specific method of random sequence generation. Most RCTs did not explicitly state how the treatment assignment, as well as the patients and researchers were blinded.

Descriptive analysis showed that TC is an effective and safe method for the treatment of EH, especially in the control of blood pressure in patients. Due to the low quality of methodology and evidence from the included studies, the conclusions of SRs/MAs may deviate from the real results, so we cannot draw firm conclusions about TC for EH.

### 4.2. Implications for Practice and Research

Previous studies have shown that TC exercise can increase the central excitability of respiration, spread the excitatory focus to the parasympathetic nerves, relax small peripheral pulses, and reduce spasticity, blood flow resistance, and blood pressure [40]. Besides, during TC exercise, patients may benefit in the following two ways: firstly, the loss amount of sodium may exceed the normal intake level [41]; secondly, the plasma nitric oxide (NO) metabolite levels are higher than normal [42], and both factors can lead to improved blood pressure.

Through a comprehensive assessment of all aspects of the included MAs using AMSTAR-2, PRISMA, ROBIS, and GRADE, it was found that the methodological and evidentiary quality was not satisfactory, which implied that there was considerable scope for addressing the quality issues in the process of conducting SRs/MAs. Researchers should register or publish research protocols in advance when conducting SRs/MAs to minimize the risk of bias and ensure the accuracy of SRs/MAs results, and they should also provide a list of excluded literature as well as explanations to ensure transparency and avoid publication bias. For literature at high risk of bias, researchers should conduct separate analyses and provide reasonable explanations to ensure the quality of the evidence. In addition, a complete assessment of publication bias would also improve the accuracy of the SRs/MAs results. Although the specificity of TC treatment may make blinding difficult, a well-designed and rigorously executed RCT is believed to be the gold standard for evaluating interventions to minimize or avoid bias [43], and future RCTs should employ a more rigorous and scientific method to solve the above problems.

TC originated from traditional Chinese medicine theory, and the duration, frequency, and mode of TC movement vary greatly in different studies. Therefore, we propose to use a standardized TC training program, including fixed duration, frequency, and pattern to better study the impact of TC on EH, which can also effectively reduce the inconsistency of SRs/MAs and enhance the credibility of the evidence. In addition, currently published SRs/MAs ignore the evaluation of blood NO and endothelin-1 levels, and the evaluation of these vascular endothelial function-related indicators can also help us to better understand the underlying mechanism of TC intervention. Future studies should complement the assessment of endothelial function by adding the assessment of circulating biochemical markers. Therefore, in future RCTs of TC interventions for EH, researchers are expected to address the issue of blinding; standardize the duration, frequency, and pattern of TC exercise; and pay attention to circulating biochemical markers so as to better explore the intrinsic mechanisms by which TC exercise exerts its efficacy.

### 4.3. Strength and Limitations

Our overview is the first to use AMSTAR2, ROBIS, PRISMA, and GRADE to evaluate SRs/MAs regarding the effect of TC on EH. The evaluation process revealed clear limitations of the current relevant SRs/MAs and RCTs, which may help guide future high-quality clinical studies. However, this overview has certain limitations because of the subjectivity of the assessment. While our assessments were assessed and reviewed by two independent assessors, different assessors may have their own judgment on each factor, so the results may vary. Although the choice of AMSTAR-2 for quality assessment is an advantage of this study, it also comes with a shortcoming, e.g., 25% of the included SRs/MAs were published before the release of AMSTAR-2, so some authors may not follow the rules, which may partly contribute to the low quality of the assessment. Besides, only one SR/MA methodology was considered to be of high quality, and therefore the evidence for the impact of TC on EH should be approached with caution.

## 5. Conclusion

In conclusion, TC is beneficial and safe for EH. However, due to the generally low quality of methodology and evidence in the included MAs, clinicians should approach this finding with caution in their practice.

## Figures and Tables

**Table 1:** Search strategy for the PubMed database.

Query	Search term
#1	“Tai Ji” [Mesh]
#2	“Tai-ji” OR “Tai Chi” OR “Chi, Tai” OR “Tai Ji Quan” OR “Ji Quan, Tai” OR “Quan, Tai Ji” OR “Taiji” OR “Taijiquan” OR “T'ai Chi” OR “Tai Chi Chuan” OR “Tai ji”
#3	#1 OR #2
#4	“Hypertension” [Mesh]
#5	“Blood Pressure, High” OR “Blood Pressures, High” OR “High Blood Pressure” OR “High Blood Pressures” OR “Hypertension”
#6	#4 OR #5
#7	Meta-Analysis as Topic [Mesh]
#8	“Systematic review” OR “meta-analysis” OR “meta analysis” OR “meta-analyses” OR “Review, Systematic”
#9	#7 OR #8
#10	#3 AND #6 AND #9

**Table 2:** Characteristics of the included SRs/MAs.

Author, year (country)	Trials (subjects)	Intervention group	Control group	Quality assessment	Main results
Zhong et al., 2020 (China) [26]	28 (2,937)	TC, TC+Control Group	HE, AHD, NT, OE	Cochrane Criteria	TC may be recommended as an adjunctive treatment for HT, especially in patients under the age of 50.
Liang et al., 2020 (China) [27]	15 (1,543)	TC, TC+Control Group	HE, AHD, NT, OE	Cochrane Criteria	TC reduces blood pressure, TCL, TG, LDL, and blood glucose, and significantly improves QOL in adult patients with EH.
Wang et al., 2013 (China) [28]	18 (1,371)	TC, TC+Control Group	AHD, RC	Cochrane Criteria	There is some encouraging evidence that TC reduces blood pressure in hypertensive patients, but the evidence remains weak due to the poor methodological quality of the included studies.
Pan et al., 2021 (China) [29]	24 (2,095)	TC, TC+Control Group	AHD, NT, OE	Cochrane Criteria	The results showed that TC exercise could effectively reduce SBP, DBP and QOL in hypertensive patients. Therefore, it should be promoted as a safe and effective adjuvant therapy for hypertension.
Song et al., 2021 (China) [30]	10 (1,177)	TC, TC+Control Group	HE, AHD, NT	Cochrane Criteria	TC can be an effective therapy for improving the QOL of patients with EH.
Guan et al., 2020 (China) [31]	13 (1,461)	TC	HE, AHD	Cochrane Criteria	Compared with the control group intervention, TC is an effective physical exercise intervention in patients with EH.
Lian et al., 2017 (China) [32]	20 (1,641)	TC, TC+Control Group	HE, AHD, NT, OE	Cochrane Criteria	The results of this study show that TC can reduce SBP, DBP, BMI, and WC.
Cai et al., 2016 (China) [33]	8 (881)	TC, TC+Control Group	AHD, NT	Cochrane Criteria	The results of this meta-analyses suggest that TC exercise can reduce SBP in patients with EH. At the same time, on the basis of conventional western medicine treatment, TC exercise can significantly lower blood pressure than the group using western medicine alone, with better curative effect and fewer side effects.
Jin et al., 2018 (China) [34]	19 (1,545)	TC	HE, AHD, NT	Jadad Scale	TC can reduce SBP and DBP to different degrees in middle-aged and elderly patients with EH. It is one of the effective methods for non-drug treatment of hypertension, and can provide a basis for the choice of clinical treatment of hypertension.
Li et al., 2011 (China) [35]	5 (402)	TC, TC+Control Group	AHD, NT, OE	Jadad Scale	TC exercise is effective in treating EH, both in reducing SBP and DBP.
Zhang et al., 2017 (China) [36]	6 (629)	TC	AHD	Jadad Scale	This study shows that TC exercise can effectively improve blood pressure in patients with EH.
Zhang et al., 2019 (China) [37]	15 (732)	TC, TC+Control Group	AHD, NT	Cochrane Criteria	The results show that TC can effectively reduce SBP and DBP in patients with EH, especially in patients under 65 years old.

**Table 3:** This overview contains the distribution table of RCTs contained in SRs/MAs.

RCT ID	Zhong et al., 2020 (China) [26]	Liang et al., 2020 (China) [27]	Wang et al., 2013 (China) [28]	Pan et al., 2021 (China) [29]	Song et al., 2021 (China) [30]	Guan et al., 2020 (China) [31]	Lian et al., 2017 (China) [32]	Cai et al., 2016 (China) [33]	Jin et al., 2018 (China) [34]	Li et al., 2011 (China) [35]	Zhang et al., 2017 (China) [36]	Zhang et al., 2019 (China) [37]	No. of times included
Sun, 2015	✔	✔		✔	✔	✔	✔	✔	✔				8
Luo, 2006	✔	✔	✔	✔			✔	✔		✔		✔	8
Zhou, 2007	✔	✔	✔	✔			✔		✔			✔	7
Zheng, 2015	✔	✔		✔	✔		✔	✔	✔				7
Qi, 2015	✔	✔		✔			✔		✔			✔	6
TSAI, 2003	✔			✔			✔	✔	✔			✔	6
Han, 2010	✔		✔	✔	✔		✔		✔				6
Mao, 2006	✔		✔				✔		✔		✔	✔	6
Xie, 2014	✔			✔			✔		✔			✔	5
Sun, 2014	✔	✔					✔		✔		✔		5
Xiao, 2018	✔	✔		✔	✔							✔	5
Xu, 2016b	✔	✔			✔	✔	✔						5
Ma, 2018	✔	✔		✔	✔	✔							5
Wang, 2011b			✔	✔					✔		✔	✔	5
Lo, 2012			✔			✔		✔	✔			✔	5
Shi, 2017	✔	✔		✔								✔	4
Shou, 2018	✔	✔		✔	✔								4
Sun, 2010	✔			✔			✔			✔			4
Liu, 2018	✔	✔		✔	✔								4
Chen, 2013	✔			✔			✔				✔		4
Tang, 2009		✔	✔						✔			✔	4
Chen, 2006			✔				✔		✔	✔			4
Lee, 2004			✔			✔		✔				✔	4
Jin, 2016	✔	✔					✔						3
Wei, 2015	✔			✔			✔						3
Chan, 2018		✔		✔		✔							3
Young, 1999				✔		✔			✔				3
Pan, 2015						✔			✔			✔	3
Wolf, 2006						✔		✔	✔				3
Zhang, 2017	✔			✔									2
Zhou, 2015	✔						✔						2
Chen, 2011a	✔		✔										2
Wang, 2019	✔			✔									2
Wang, 2011a			✔				✔						2
He, 2011			✔						✔				2
Wang, 2007a			✔							✔			2
Wang, 2007b			✔							✔			2
Kim, 2016				✔								✔	2
Kim, 2014				✔								✔	2
Tang, 2008				✔			✔						2
Thomas, 2005						✔		✔					2
Lu, 2015									✔		✔		2
Lin, 2019	✔												1
Liu, 2017	✔												1
Chen, 2011b			✔										1
Xu, 2016a							✔						1
Li, 2016	✔												1
Pan, 2014	✔												1
Wei, 2003			✔										1
Yi, 1990			✔										1
Song, 2011			✔										1
Gou, 2017					✔								1
Li, 2018					✔								1
Lee, 2017						✔							1
Nguyen, 2012						✔							1
Hsu, 2015						✔							1
Jing, 2015									✔				1
Zheng, 2014											✔		1
Total studies included	28	15	18	24	10	13	20	8	19	5	6	15	

**Table 4:** Result of the AMSTAR-2 assessments.

Author, year (country)	Q1	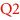	Q3	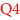	Q5	Q6	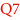	Q8	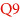	Q10	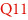	Q12	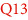	Q14	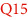	Q16	Quality
Zhong, et al., 2020 (China) [26]	Y	Y	Y	Y	Y	Y	Y	Y	Y	Y	Y	Y	Y	N	Y	Y	H
Liang et al., 2020 (China) [27]	Y	Y	Y	Y	Y	Y	N	Y	Y	Y	Y	Y	N	Y	Y	Y	VL
Wang et al., 2013 (China) [28]	Y	PY	Y	Y	Y	Y	N	Y	Y	Y	Y	Y	Y	N	N	Y	VL
Pan et al., 2021 (China) [29]	Y	PY	Y	PY	Y	Y	N	Y	Y	Y	Y	Y	Y	Y	Y	Y	VL
Song et al., 2021 (China) [30]	Y	Y	Y	Y	Y	Y	N	Y	Y	Y	Y	Y	N	Y	N	Y	VL
Guan et al., 2020 (China) [31]	Y	PY	Y	PY	Y	Y	N	Y	Y	Y	Y	Y	N	Y	Y	Y	VL
Ziyu Lian, 2017 (China) [32]	Y	PY	Y	Y	Y	Y	N	Y	Y	Y	Y	Y	N	Y	Y	Y	VL
Cai et al., 2016 (China) [33]	Y	PY	Y	Y	N	N	N	Y	Y	Y	Y	N	N	N	N	N	VL
Chengji Jin, 2018 (China) [34]	Y	PY	Y	PY	Y	Y	N	Y	Y	Y	Y	Y	N	Y	Y	N	VL
Li et al., 2011 (China) [35]	Y	PY	Y	PY	N	N	N	Y	Y	Y	Y	Y	N	N	N	N	VL
Zhang et al., 2017 (China) [36]	Y	PY	Y	PY	N	N	N	Y	Y	Y	Y	Y	Y	Y	N	Y	VL
Zhang et al., 2019 (China) [37]	Y	PY	Y	PY	Y	Y	N	Y	Y	Y	Y	Y	N	Y	Y	N	VL

Note: Y, yes; PY, partial yes; N, no; VL, very low; H, high. Critical areas are marked in red.

**Table 5:** Results of the ROBIS assessments.

Author, year (country)	Phase 1	Phase 2				Phase 3
	Assessing relevance	Domain 1: Study eligibility criteria	Domain 2: Identification and selection of studies	Domain 3: Collection and study appraisal	Domain 4: Synthesis and findings	Risk of bias in the review
Dongling Zhong, 2020 (China) [26]	✓	✓	✓	✓	✓	✓
Hao Liang, 2020 (China) [27]	✓	✓	✓	✓	✕	✕
Jie Wang, 2013 (China) [28]	✓	✓	✓	✕	✕	✕
Xiandu Pan, 2021 (China) [29]	✓	✓	✕	✓	✓	✕
Yang Song, 2021 (China) [30]	✓	✓	✓	✓	✕	✕
Yuanyuan Guan, 2020 (China) [31]	✓	✓	✕	✕	✕	✕
Ziyu Lian, 2017 (China) [32]	✓	✓	✓	✓	✕	✕
Lu Cai, 2016 (China) [33]	✓	✓	✓	✕	✕	✕
Chengji Jin, 2018 (China) [34]	✓	✓	✕	✓	✓	✕
Hongguo Li, 2011 (China) [35]	✓	✓	✕	✕	✕	✕
Yeting Zhang, 2017 (China) [36]	✓	✓	✕	✕	✕	✕
Yongpeng Zhang, 2019 (China) [37]	✓	✓	✕	✓	✕	✕

Note:✔, low risk; ✕, high risk.

**Table 6:** Results of the PRISMA checklist.

Section/topic	Items	Dongling Zhong, 2020 (China) [26]	Hao Liang, 2020 (China) [27]	Jie Wang, 2013 (China) [28]	Xiandu Pan, 2021 (China) [29]	Yang Song, 2021 (China) [30]	Yuanyuan Guan, 2020 (China) [31]	Ziyu Lian, 2017 (China) [32]	Lu Cai, 2016 (China) [33]	Chengji Jin, 2018 (China) [34]	Hongguo Li, 2011 (China) [35]	Yeting Zhang, 2017 (China) [36]	Yongpeng Zhang, 2019 (China) [37]	Number of yes (%)
Title	Q1. Title	Y	Y	Y	Y	Y	Y	Y	Y	Y	Y	Y	Y	100
Abstract	Q2. Structured summary	Y	Y	Y	Y	Y	Y	Y	Y	Y	Y	Y	Y	100
Introduction	Q3. Rationale	Y	Y	Y	Y	Y	Y	Y	Y	Y	Y	Y	Y	100
	Q4. Objectives	Y	Y	Y	Y	Y	Y	Y	Y	Y	Y	Y	Y	100
Methods	Q5. Protocol and registration	Y	Y	N	N	Y	N	N	N	N	N	N	N	25
	Q6. Eligibility criteria	Y	Y	Y	Y	Y	Y	Y	Y	Y	Y	Y	Y	100
	Q7. Information sources	Y	Y	Y	Y	Y	Y	Y	Y	Y	Y	Y	Y	100
	Q8. Search	Y	Y	N	N	N	N	N	N	N	N	N	N	16.67
	Q9. Study selection	Y	Y	Y	Y	Y	Y	Y	Y	Y	Y	Y	Y	100
	Q10. Data collection process	Y	Y	Y	Y	Y	Y	Y	Y	Y	Y	Y	Y	100
	Q11. Data items	Y	Y	Y	Y	Y	Y	Y	Y	Y	Y	Y	Y	100
	Q12. Risk of bias in individual studies	Y	Y	Y	Y	Y	Y	Y	Y	Y	Y	Y	Y	100
	Q13. Summary measures	Y	Y	Y	Y	Y	Y	Y	Y	Y	Y	Y	Y	100
	Q14. Synthesis of results	Y	Y	N	Y	Y	Y	Y	Y	Y	Y	Y	Y	91.67
	Q15. Risk of bias across studies	Y	Y	Y	Y	Y	Y	Y	N	Y	N	Y	Y	83.33
	Q16. Additional analyses	Y	Y	N	Y	N	N	Y	N	Y	N	N	Y	50
Results	Q17. Study selection	Y	Y	Y	Y	Y	Y	Y	Y	Y	Y	Y	Y	100
	Q18. Study characteristics	Y	Y	Y	Y	Y	Y	Y	Y	Y	Y	Y	Y	100
	Q19. Risk of bias within studies	Y	Y	Y	Y	Y	Y	Y	Y	Y	Y	Y	Y	100
	Q20. Results of individual studies	Y	Y	Y	Y	Y	Y	Y	Y	Y	Y	Y	Y	100
	Q21. Synthesis of results	Y	Y	N	Y	Y	Y	Y	Y	Y	Y	Y	Y	91.67
	Q22. Risk of bias across studies	Y	Y	N	Y	N	Y	Y	N	Y	N	N	Y	58.33
	Q23. Additional analysis	Y	Y	Y	Y	Y	Y	N	N	Y	N	N	Y	66.67
Discussion	Q24. Summary of evidence	Y	Y	Y	Y	Y	Y	Y	Y	Y	Y	Y	Y	100
	Q25. Limitations	Y	Y	Y	Y	Y	Y	Y	Y	Y	Y	Y	Y	100
	Q26. Conclusions	Y	Y	Y	Y	Y	Y	Y	Y	Y	Y	Y	Y	100
Funding	Q27. Funding	Y	Y	Y	Y	Y	Y	Y	N	N	N	Y	N	66.67

Note: Y, yes; N, no.

**Table 7:** Results of evidence quality.

Author, year (country)	Outcomes	Studies (participants)	Limitations	Inconsistency	Indirectness	Imprecision	Publication bias	Relative effect (95% CI)	Quality
Dongling Zhong, 2020 (China)	SBP (TC vs. HE/NT)	9 (974)	−1①	−1②	0	0	−1④	MD = −14.784 (−19.587, −9.981)^*∗*^	Very low
	DBP (TC vs. HE/NT)	9 (974)	−1①	0	0	0	−1④	MD = −7.035 (−9.083, −4.988)^*∗*^	Low
	SBP (TC vs. OE)	5 (352)	−1①	−1②	0	−1③	−1④	MD = −7.934 (−14.221, −1.674)^*∗*^	Very low
	DBP (TC vs. OE)	5 (352)	−1①	0	0	−1③	−1④	MD = −3.856 (−6.544, −1.168)^*∗*^	Very low
	SBP (TC vs. AHD)	15 (1,508)	−1①	−1②	0	0	0	MD = −9.070 (−14.033, −4.108)^*∗*^	Low
	DBP (TC vs. AHD)	15 (1,508)	−1①	−1②	0	0	−1④	MD = −5.625 (−8.836, −2.414)^*∗*^	Very low
	TCL (TC vs. HE/NT)	3 (362)	−1①	0	0	0	−1④	MD = −0.753 (−1.161, −0.345)^*∗*^	Low
	TG (TC vs. HE/NT)	3 (362)	−1①	−1②	0	−1③	−1④	MD = −0.373 (−0.795, 0.049)	Very low
	HDL (TC vs. HE/NT)	3 (362)	−1①	−1②	0	−1③	−1④	MD = 0.269 (−0.184, 0.722)	Very low
	LDL (TC vs. HE/NT)	3 (362)	−1①	−1②	0	−1③	−1④	MD = −1.048 (−1.650, −0.447)^*∗*^	Very low
	TG (TC vs. AHD)	4 (365)	−1①	−1②	0	−1③	−1④	MD = −2.238 (−3.889, −0.587)^*∗*^	Very low
Hao Liang, 2020 (China)	SBP	15 (1,543)	−1①	0	0	0	0	MD = −12.47 (−16.00, −8.94)^*∗*^	Moderate
	DBP	15 (1,543)	−1①	−1②	0	0	0	MD = −6.46 (−8.28, −4.64)^*∗*^	Low
	QOL	7 (955)	−1①	−1②	0	0	0	SMD = 0.62 (0.35, 0.90)^*∗*^	Low
	TCL	5 (846)	−1①	0	0	0	0	MD = −0.49 (−0.62, −0.37)^*∗*^	Moderate
	TG	5 (846)	−1①	−1②	0	0	0	MD = −0.49 (−0.92, −0.07)^*∗*^	Low
	LDL	5 (846)	−1①	−1②	0	0	0	MD = −0.86 (−1.30, −0.43)^*∗*^	Low
	HDL	5 (846)	−1①	−1②	0	−1③	0	MD = −0.92 (−2.21, 0.37)	Very low
	Blood glucose	4 (612)	−1①	−1②	0	0	−1⑤	MD = −0.91 (−1.59, −0.23)^*∗*^	Very low
Jie Wang, 2013 (China)	Efficient (TC vs. RC)	4 (220)	−1①	−1②	0	−1③	−1④	RR = 3.39 (1.81, 6.34)^*∗*^	Very low
	SBP (TC vs.RC)	10 (896)	−1①	−1②	0	0	−1④	MD = −12.43 (−12.62,−12.24)^*∗*^	Very low
	SBP (TC +AHDvs. AHD)	2 (72)	−1①	−1②	0	−1③	−1④	MD = −9.34 (−10.89, −7.79)^*∗*^	Very low
	DBP (TC vs.RC)	10 (896)	−1①	−1②	0	0	−1④	MD = −6.03 (−6.16，−5.90)^*∗*^	Very low
	DBP (TC +AHDvs. AHD)	2 (72)	−1①	−1②	0	−1③	−1④	MD = −7.16 (−7.71, −6.60)^*∗*^	Very low
Xiandu Pan, 2021 (China)	SBP	24 (2,107)	0	−1②	0	0	0	SMD = −1.05 ( −1.44, −0.67)^*∗*^	Moderate
	DBP	24 (2,107)	0	−1②	0	0	0	SMD = −0.91 (−1.24, −0.58)^*∗*^	Moderate
	BMI	6 (790)	0	0	0	0	−1④	SMD = −0.08 ( −0.35, −0.19)^*∗*^	Moderate
	Physical function	7 (853)	0	−1②	0	0	−1④	SMD = 0.86 (0.36, 1.37)^*∗*^	Low
	Role physical	7 (853)	0	0	0	0	−1④	SMD = 0.86 (0.61, 1.11)^*∗*^	Moderate
	General health	7 (853)	0	−1②	0	0	−1④	SMD = 0.75 (0.32, 1.17)^*∗*^	Low
	Bodily pain	7 (853)	0	−1②	0	0	−1④	SMD = 0.65 (0.29, 1.00)^*∗*^	Low
	Vitality	7 (853)	0	−1②	0	0	−1④	SMD = 0.71 (0.34, 1.07)^*∗*^	Low
	Social function	7 (853)	0	−1②	0	0	−1④	SMD = 0.63 (0.07, 1.19)^*∗*^	Low
	Role emotional	7 (853)	0	−1②	0	0	−1④	SMD = 0.64 (0.22, 1.06)^*∗*^	Low
	Mental health	7 (853)	0	−1②	0	0	−1④	SMD = 0.73 (0.31, 1.16)^*∗*^	Low
Yang Song, 2021 (China)	Physical function	8 (981)	0	0	0	0	−1④	MD = 7.54 (5.65, 9.43)^*∗*^	Moderate
	Role physical	8 (981)	0	−1②	0	0	−1④	MD = 10.07 (6.64, 13.49)^*∗*^	Low
	Bodily pain	7 (859)	0	−1②	0	0	−1④	MD = 9.40 (4.67, 14.13)^*∗*^	Low
	General health	8 (981)	0	−1②	0	0	−1④	MD = 6.95 (2.51, 11.39)^*∗*^	Low
	Vitality	7 (859)	0	0	0	0	−1④	MD = 9.40 (7.87, 10.93)^*∗*^	Moderate
	Social function	7 (859)	0	−1②	0	0	−1④	MD = 9.56 (2.84, 16.28)^*∗*^	Low
	Role emotional	7 (859)	0	−1②	0	0	−1④	MD = 9.09 (3.62, 14.55)^*∗*^	Low
	Mental health	8 (981)	0	0	0	0	−1④	MD = 9.85 (7.08, 12.61)^*∗*^	Moderate
Yuanyuan Guan, 2020 (China)	SBP	13 (1,461)	−1①	0	0	0	0	MD = −6.58 (−8.14, −5.02)^*∗*^	Moderate
	DBP	13 (1,461)	−1①	0	0	0	0	SMD = −0.57 (−0.77, −0.37)^*∗*^	Moderate
	TCL	4 (476)	−1①	−1②	0	0	0	SMD = −0.29, (−0.73, 0.15)	Low
	TG	3 (448)	0	0	0	0	0	SMD = −0.19, (−0.22, −0.16)^*∗*^	High
	HDL	4 (612)	0	−1②	0	−1③	0	SMD = 0.59, (−0.12, 1.29)	Low
	LDL	3 (448)	0	0	0	0	0	SMD = −12.55, (−15.96, −9.14)^*∗*^	High
	BMI	7 (1,039)	−1①	−1②	0	−1③	0	SMD = −0.11, (−0.75, 0.52)	Very low
	WC	4 (638)	0	0	0	0	0	SMD = −0.37, (−0.63, −0.10)^*∗*^	High
Ziyu Lian, 2017 (China)	DBP(TC vs. NT)	10 (875)	−1①	0	0	0	0	SMD = −0.84, (−1.18, −0.50)^*∗*^	Moderate
	BMI(TC vs. NT)	4 (451)	−1①	0	0	0	−1④	SMD = −0.39, (−0.73, −0.06)^*∗*^	Low
	WC(TC vs. NT)	3 (375)	−1①	0	0	0	−1④	SMD = −0.53, (−0.74, −0.32)^*∗*^	Low
	SBP(TC vs. AHD)	3 (210)	0	−1②	0	−1③	0	SMD = −0.81, (−1.40, −0.22)^*∗*^	Low
	DBP(TC vs. AHD)	3 (210)	0	−1②	0	−1③	0	SMD = −0.75, (−1.60, −0.10)^*∗*^	Low
Lu Cai, 2016 (China)	SBP(TC vs. HE/NT)	6 (881)	0	−1②	0	0	−1④	MD = −9.56 (−15.29, −3.82)^*∗*^	Low
	DBP(TC vs. HE/NT)	6 (881)	0	−1②	0	−1③	−1④	MD = −4.79（−9.83，0.26)	Very Low
	SBP(TC +AHDvs. AHD)	2 (182)	−1①	0	0	−1③	−1④	MD = −13.97 （−16.73，−11.22)^*∗*^	Very low
	DBP(TC +AHDvs. AHD)	2 (182)	−1①	0	0	−1③	−1④	MD = −10.31 （−12.15，−8.46)^*∗*^	Very low
Chengji Jin, 2018 (China)	SBP	19 (1,545)	0	−1②	0	0	0	MD = 11.14 (7.82,14.47)^*∗*^	Moderate
	DBP	19 (1,545)	0	−1②	0	0	0	MD = 5.64 (3.34,7.94)^*∗*^	Moderate
Hongguo Li, 2011 (China)	SBP	2 (104)	0	−1②	0	−1③	−1④	MD = 18.93 (8.16,29.71)^*∗*^	Very low
	DBP	2 (104)	0	0	0	−1③	−1④	MD = 8.95 (5.61，12.3)^*∗*^	Low
	Efficient	4 (298)	−1①	0	0	−1③	−1④	OR = 4.59 (2.55, 8.24)^*∗*^	Very low
Yeting Zhang, 2017 (China)	SBP	6 (434)	0	0	0	0	−1④	MD = 14.30 (11.74, 16.86)^*∗*^	Moderate
	DBP	5 (354)	0	0	0	−1③	−1④	MD = 5.48 (4.07, 6.90)^*∗*^	Low
Yongpeng Zhang, 2019 (China)	SBP	15 (732)	−1①	−1②	0	0	0	SMD = 1.22(1.07, 1.37)^*∗*^	Low
	DBP	15 (732)	−1①	−1②	0	0	−1④	SMD = 0.63(0.49, 0.77)^*∗*^	Very low

Note: ①The included studies had a large bias in methodology such as randomization, allocation concealment, and blinding. ②The confidence interval overlapped less or the *I*^2^ value of the combined results was larger. ③The sample size from the included studies did not meet the optimal sample size or the 95% confidence interval crossed the invalid line. ④The funnel chart was asymmetry. ⑤Few studies were included, and their results were all positive, which may result in a large publication bias. ^*∗*^The 95% confidence interval did not cross the invalid line.

## Data Availability

The datasets analyzed during the current study are available from the corresponding author on reasonable request.
